# Supplementary material for: Oncogenic virus hijacks SOX18 pioneer function to enhance viral persistence
Source: Res Sq. 2025 Aug 18:rs.3.rs-7206339. Preprint. [Version 1] doi: 10.21203/rs.3.rs-7206339/v1 (PMC12393484; doi:10.21203/rs.3.rs-7206339/v1)
Supplement: 1 [file NIHPPRS7206339V1-supplement-1.pdf]

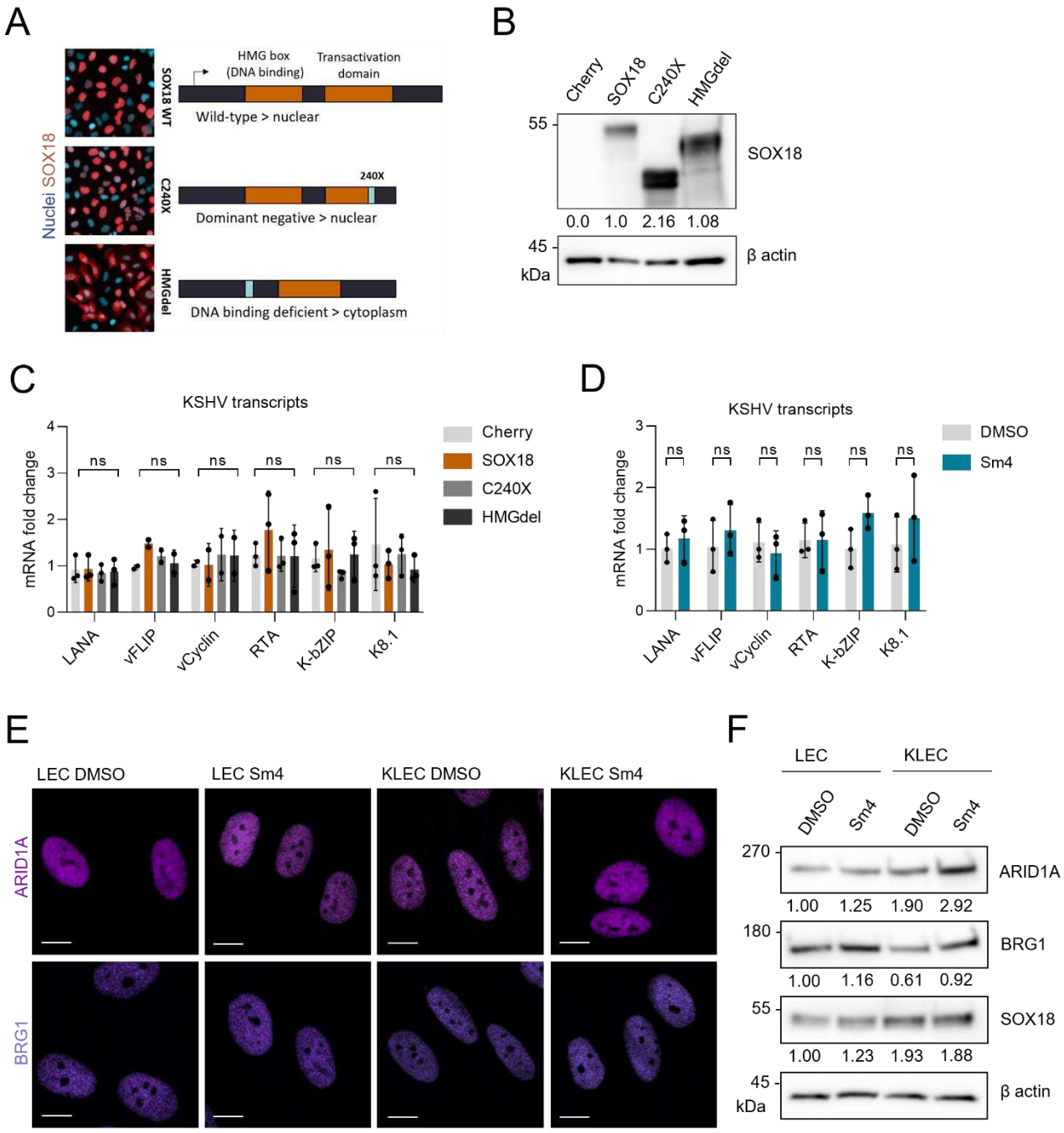
A
Nuclei SOX18
SOX18 WT
HMG box (DNA binding)
Transactivation domain
Wild-type > nuclear
C240X
240X
Dominant negative > nuclear
HMGdel
DNA binding deficient > cytoplasm
B
Cherry
SOX18
C240X
HMGdel
55
SOX18
0.0 1.0 2.16 1.08
45
kDa
β actin
C
KSHV transcripts
mRNA fold change
ns
LANA
vFLIP
vCyclin
RTA
K-bZIP
K8.1
Cherry
SOX18
C240X
HMGdel
D
KSHV transcripts
mRNA fold change
DMSO
Sm4
E
LEC DMSO
LEC Sm4
KLEC DMSO
KLEC Sm4
ARID1A
BRG1
F
LEC
KLEC
DMSO
Sm4
270
ARID1A
1.00 1.25 1.90 2.92
180
BRG1
1.00 1.16 0.61 0.92
55
SOX18
1.00 1.23 1.93 1.88
45
kDa
β actin

Supplementary Figure 1. Related to Fig 1.

A-C. HeLa cells expressing SOX18wt, mutants C240X (dominant-negative transactivation deficient) or HMGdel (DNA-binding deficient), or mCherry as a control, and thereafter infected with rKSHV.219 for 72h (KSHV-HeLa). **A.** IF images of the SOX18wt and mutants expressing cells labeled with anti-SOX18 antibody and a schematic of the constructs. **B.** Immunoblotting with anti-SOX18 antibody using β-actin as a loading control for normalization. **C.** RT-qPCR for the indicated viral genes in KSHV-HeLa. **D.** LECs infected with rKSHV.219 (KLECs) for 72 hours and treated with Sm4 or DMSO control for 24h and relative mRNA measured for indicated viral transcripts. Statistical significance was determined by one-way ANOVA with Dunnett correction for multiple comparisons; ns = non-significant. **E-F.** Uninfected LECs and KLECs 72 h.p.i. treated with DMSO or Sm4 for another 72h and E) labeled with anti-ARID1A and -BRG1 antibodies, nuclei were counterstained with Hoechst (33342), scale bar is 10μm, and F) immunoblotted for the indicated proteins and quantified as in B.

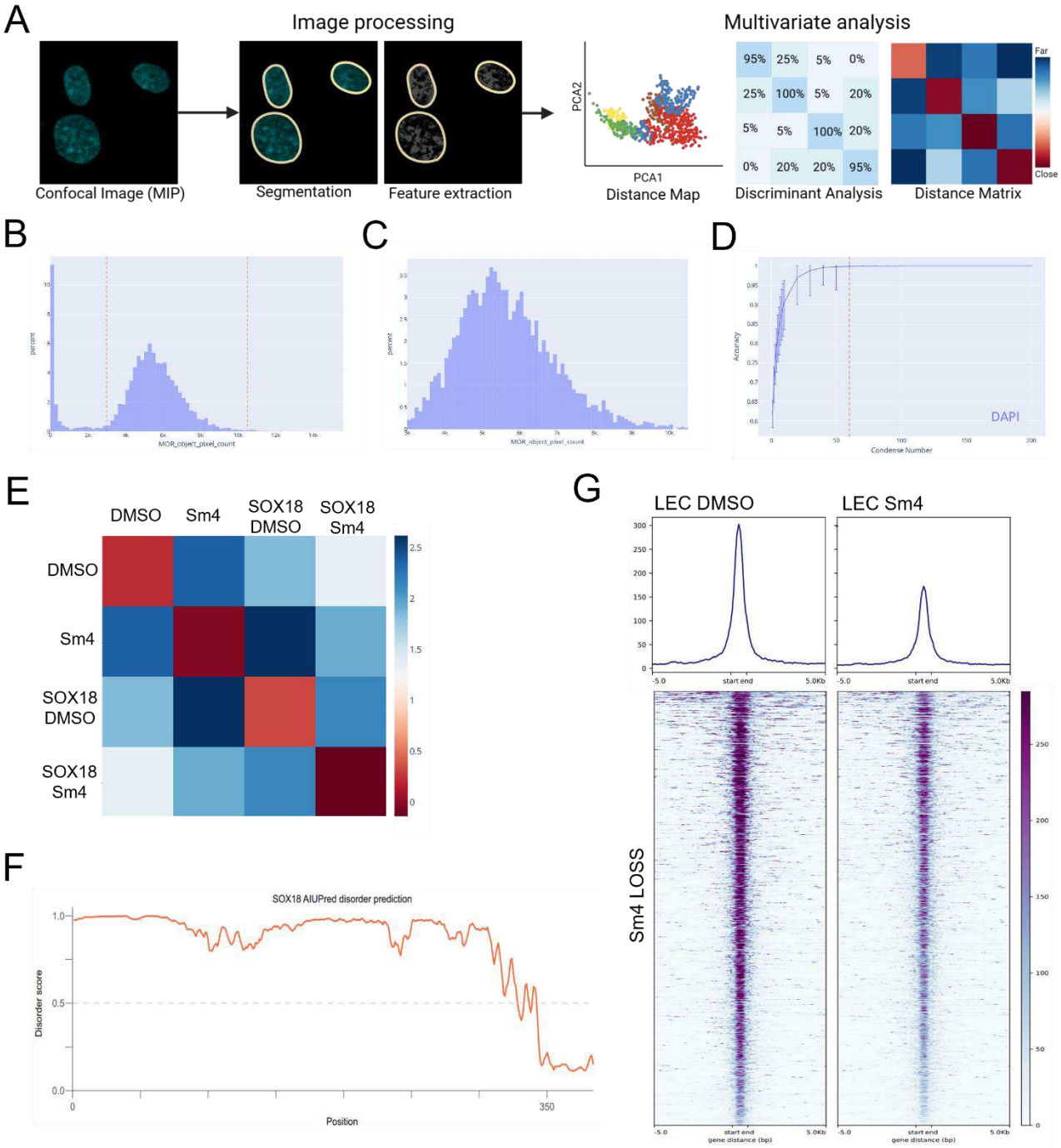
A
Image processing
Multivariate analysis
Confocal Image (MIP)
Segmentation
Feature extraction
PCA2
PCA1
Distance Map
95% 25% 5% 0%
25% 100% 5% 20%
5% 5% 100% 20%
0% 20% 20% 95%
Discriminant Analysis
Far
Close
Distance Matrix
B
C
D
DAPI
Condense Number
E
DMSO
Sm4
SOX18 DMSO
SOX18 Sm4
F
SOX18 AIUPred disorder prediction
Disorder score
Position
G
LEC DMSO
LEC Sm4
Sm4 LOSS
start end
5.0Kb
gene distance (bp)

Supplementary Figure 2. Related to Fig 2.

A. Representative pipeline of unsupervised MIEL analysis. For each experimental condition, confocal images of the total cell population are acquired and separated by fluorescence channel. Individual nuclei are segmented using DAPI staining as a mask. Within each segmented nucleus, 253 texture- and edge-based fluorescent features are extracted at the pixel level. These features are computed using the intensity relationships between each pixel and its surrounding neighbors, capturing spatial variation in signal distribution. The variation in the extracted nuclear features is quantified across all nuclei, and dimensionality reduction is performed using Principal Component Analysis (PCA). The top two principal components are used to visualize data structure and spread in a 2D PCA plot. To classify cell populations, a Support Vector Machine (SVM) algorithm is applied, enabling the identification of distinct clusters based on feature signatures. The average pairwise distances points in PCA space are then computed and represented as a similarity matrix. **B-C.** HUVECs ± SOX18 over-expression and ± Sm4 treatment stained with DAPI. **B.** Histogram displaying distribution of all object sizes (pixels) identified during segmentation, objects inside of red-dashed lines are used in analysis and **C.** zoom in on dashed lines in right panel. **D.** Line plots show accuracy measurements versus cell condense number; 95% confidence intervals are shown with red dotted line denotes smallest condense number above 95% accuracy. **E.** Average distance matrix calculated from the distance between each point per condition, with blue as farthest distances and red as closest distances. **F.** Intrinsic disorder prediction using AIUPred for SOX18 transcription factor (Uniprot ID: P35713). Y-axis represents the disorder prediction score and x-axis amino acid position. Scores over 0.5 (dashed line) are considered disordered. **G.** LECs treated with DMSO or Sm4 and subjected to ATAC-seq. Heatmap showing chromatin accessibility loss on the top 1000 sites of host genome (dark blue maps and line) upon Sm4 treatment.

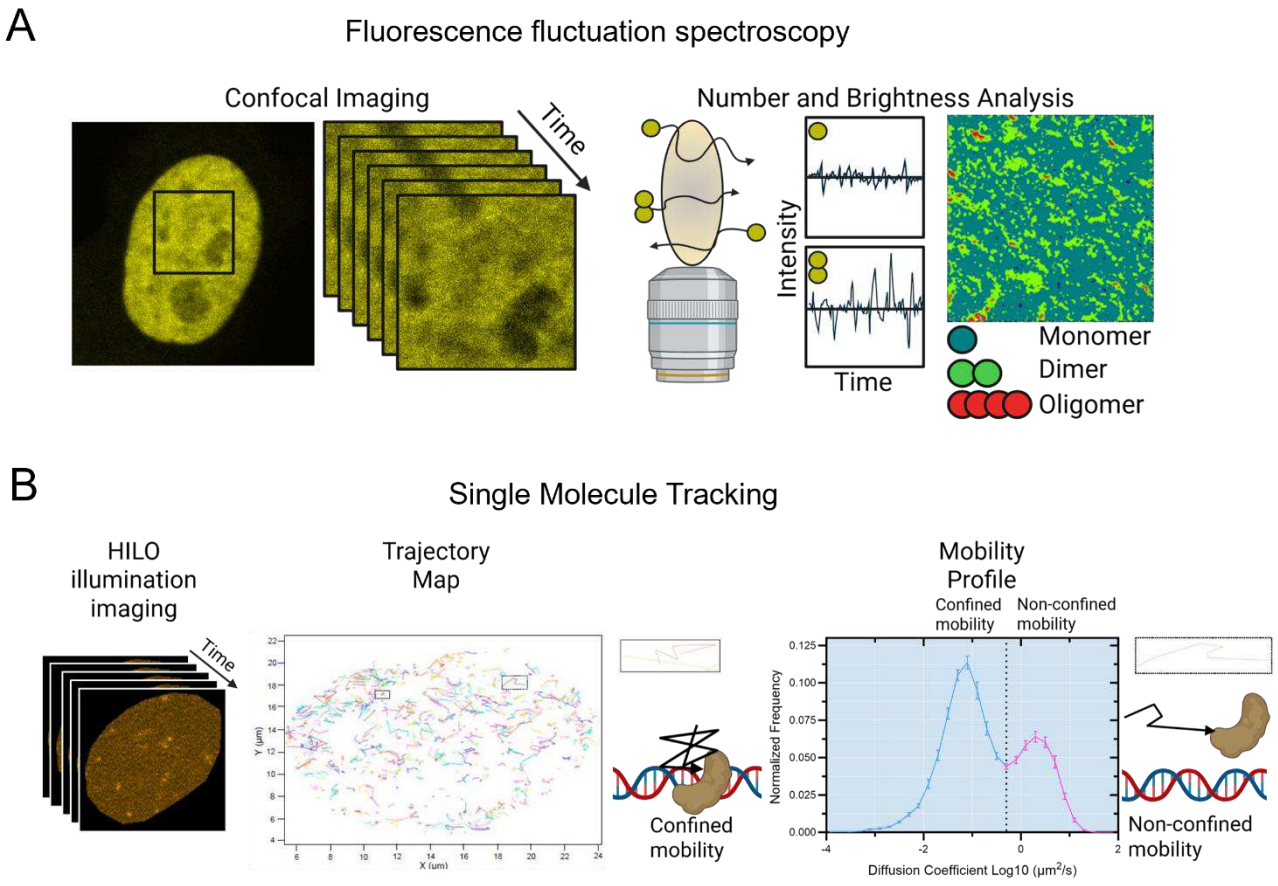

1484 **Supplementary Figure 3. Related to Fig 3.**

1485 **A.** Number and Brightness (N&B) analysis starts by identifying a cell and raster scanning to generate
1486 a confocal time series (100 frames). As the molecules move through the confocal volume the different
1487 oligomeric states will cause differences in fluorescence intensity. The intensity fluctuations are
1488 assessed over time and converted to fluctuations in molecular brightness for every pixel. The
1489 brightness is indicative of the average oligomeric state. In this way a dimer is twice as bright as a
1490 monomer and higher order oligomer are brighter than a dimer. **B.** Single molecule tracking (SMT)
1491 analysis starts by identifying a cell and imaging by highly inclined and laminated optical sheet (HILO)
1492 illumination to generate a time series (6000 frames). From the time series each Halo-tagged SOX18
1493 molecule is identified per frame and stitched together to build a trajectory map. From each trajectory
1494 the diffusion coefficient is calculated as a measure of molecular mobility. Trajectories that have a low
1495 diffusion coefficient are defined as having a confined mobility (blue), whereas trajectories that have a
1496 higher diffusion coefficient are defined as being diffusive (non-confined mobility; pink). The diffusion
1497 coefficients are then graphed to assess the proportion of molecular populations that falls into either
1498 category.

SOX18 mobility and oligomeric state is effected by chromatin organization			
**N&B (=PPI, oliogmeric status)**	**Biophysical Observation**	**Biologlical interpretation**	**Fig**
ActD treatment relative to DMSO	No significant difference in the percentage of monomers and dimers, significant increase in oligomers	ActD treatment = loss of chromatin accessibility, leading to the loss of binding locations and subsequent decline in dimer formation.	Fig 3A-D
TSA treatment relative to DMSO	Significant increase in percentage of monomers & significant decrease in percentage of dimers, no significant difference in percentage of oligomers	TSA treatment = increase in chromatin accessibility, leading to increased binding locations and an increase in the dimer and higher order oligomer formation	Fig 3A-D
**SMT (Diffusion)**	**Biophysical Observation**	**Biologlical interpretation**	**Fig**
TSA treatment relative to DMSO: non-confined mobility (= target search pattern)	Significantly decreases	Increased chromatin accessiblity from TSA = more SOX18 molecules binding and less diffusing	Fig 3E-F
TSA treatment relative to DMSO: confined mobility (= PPI or protein-chromatin interaction)	Significantly increases		Fig 3E-F
**SMT (Temporal occupancy)**	**Biophysical Observation**	**Biologlical interpretation**	**Fig**
TSA treatment relative to DMSO: short occupancy (= searching behavior)	No significant differences	Increased chromatin accessiblity from TSA = SOX18 forming more dimers and higher order oligomers	Fig 3G-I
TSA treatment relative to DMSO: long occupancy (= PPI or protein-chromatin interaction)	Signficantly increases		Fig 3G-I
TSA treatment relative to DMSO: ratio of occupancy	No signifcant differences		Fig 3G-I

Table S1. Summary of biophysical experiments and biological interpretations.

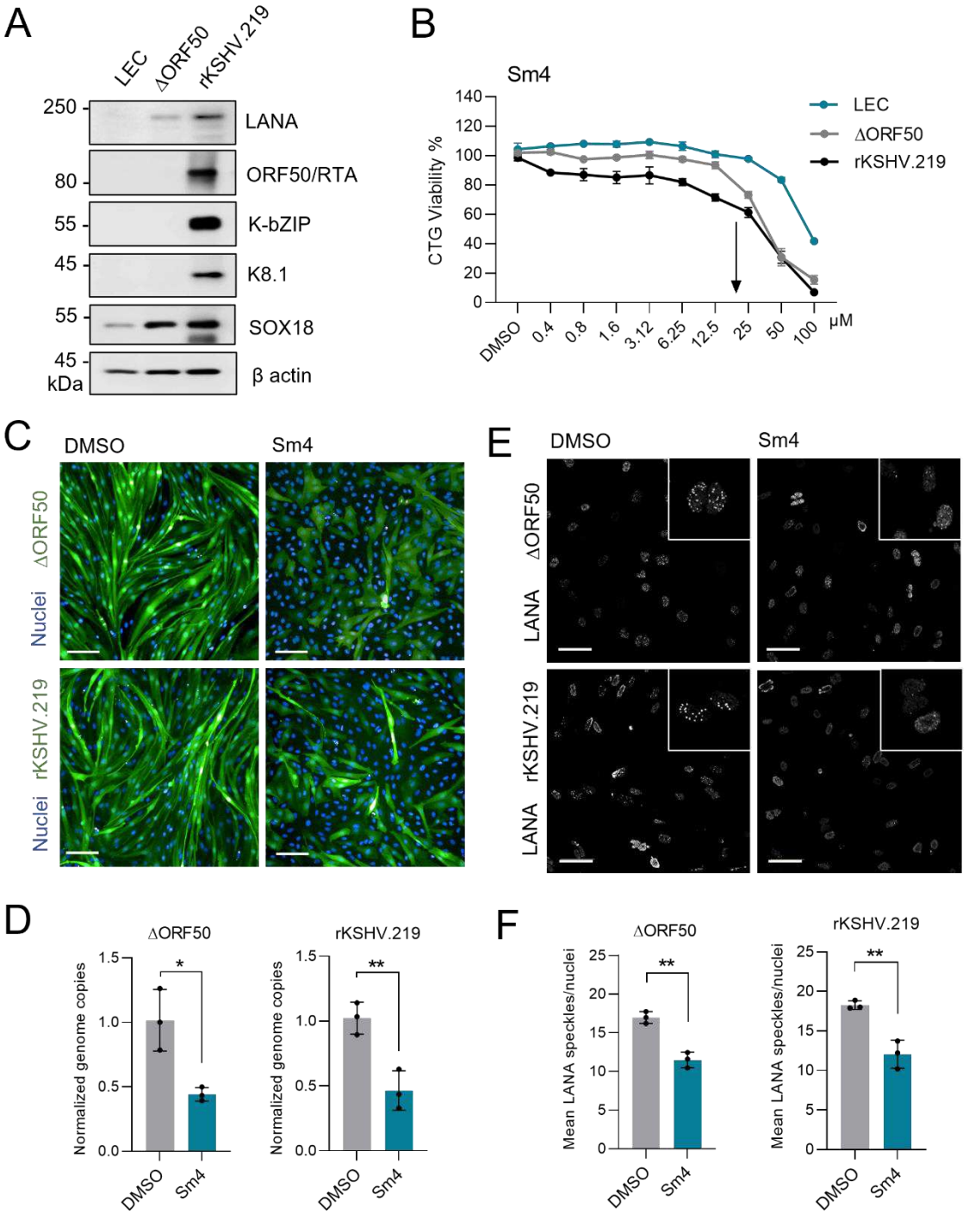
A
LEC
ΔORF50
rKSHV.219
250
80
55
45
55
45
kDa
LANA
ORF50/RTA
K-bZIP
K8.1
SOX18
β actin
B
Sm4
CTG Viability %
140
120
100
80
60
40
20
0
DMSO
0.4
0.8
1.6
3.12
6.25
12.5
25
50
100
μM
LEC
ΔORF50
rKSHV.219
C
DMSO
Sm4
ΔORF50
Nuclei
rKSHV.219
Nuclei
E
DMSO
Sm4
ΔORF50
LANA
rKSHV.219
LANA
D
ΔORF50
rKSHV.219
Normalized genome copies
1.5
1.0
0.5
0.0
*
**
DMSO
Sm4
F
ΔORF50
rKSHV.219
Mean LANA speckles/nuclei
25
20
15
10
5
0
**
DMSO
Sm4

Supplementary Figure 4. Related to Fig 4.

A. LECs infected with latent KSHV-BAC16-ΔORF50 (ΔORF50-KLEC) or wildtype rKSHV.219 (rKSHV.219) for 72h and immunoblotted for the indicated viral proteins, and SOX18, using β-actin as a loading control. **B.** CTG viability assay of LECs infected with ΔORF50 or rKSHV.219 and treated with DMSO or with the indicated increasing Sm4 concentrations. **C-F.** Infection phenotypes of LECs infected with GFP-expressing ΔORF50-KLEC or rKSHV.219 and treated at 72h.p.i with Sm4 or DMSO control for 72h. **C.** GFP images of infected cells upon DMSO or Sm4 treatments. Nuclei were counterstained with Hoechst (33342), scale bar is 100µm. **D.** Relative KSHV DNA genome copies. **E.** Images of anti-LANA labeled infected cells and F) quantified as mean from 10 fields for each n=3 biological replicates. Nuclei were counterstained with Hoechst (33342), scale bar is 50µm. Statistical significance was determined by unpaired t-test, *$p < 0.05$, **$p < 0.01$.

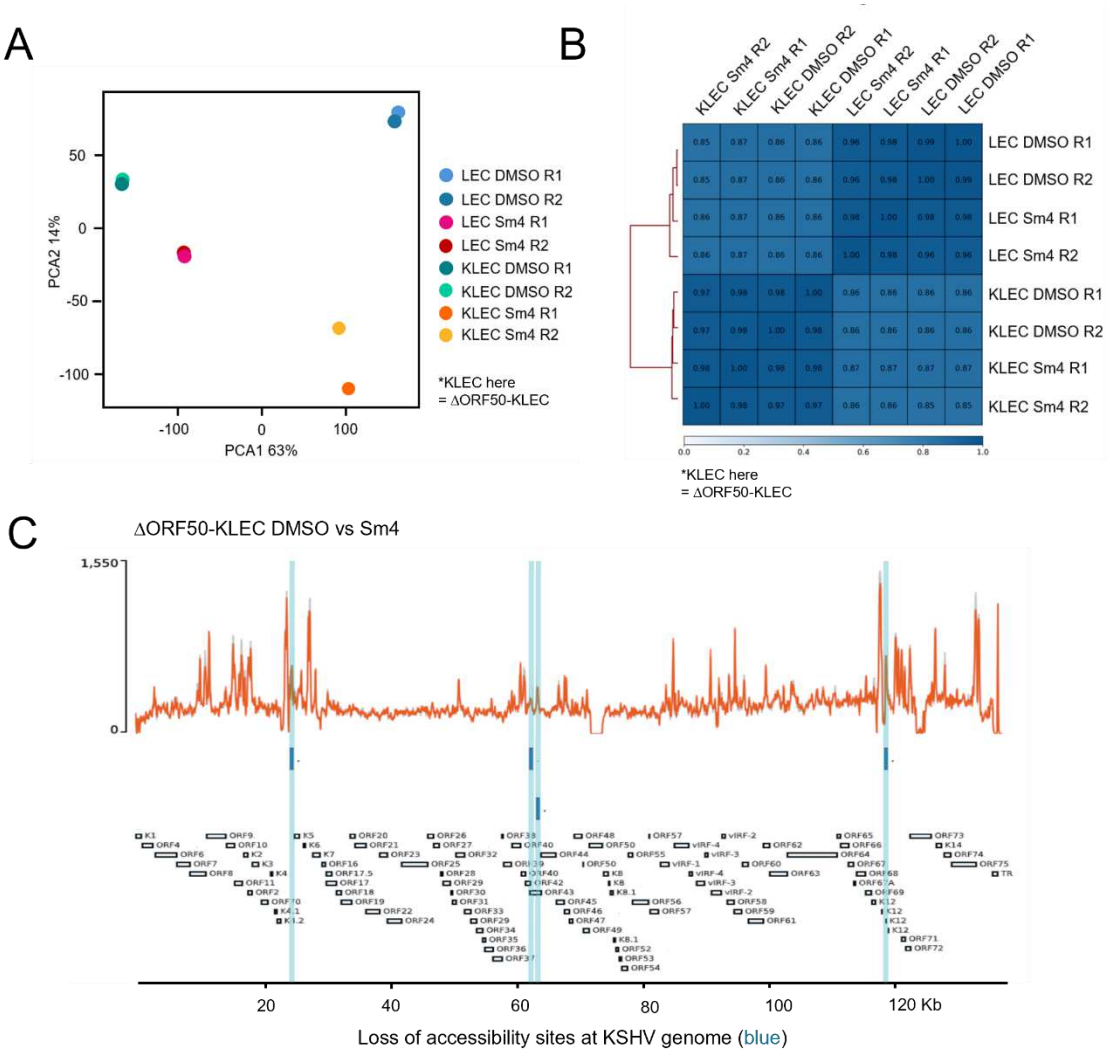
A
B
LEC DMSO R1
LEC DMSO R2
LEC Sm4 R1
LEC Sm4 R2
KLEC DMSO R1
KLEC DMSO R2
KLEC Sm4 R1
KLEC Sm4 R2
*KLEC here
= ΔORF50-KLEC
PCA1 63%
PCA2 14%
C
ΔORF50-KLEC DMSO vs Sm4
1,550
0
20
40
60
80
100
120 Kb
Loss of accessibility sites at KSHV genome (blue)

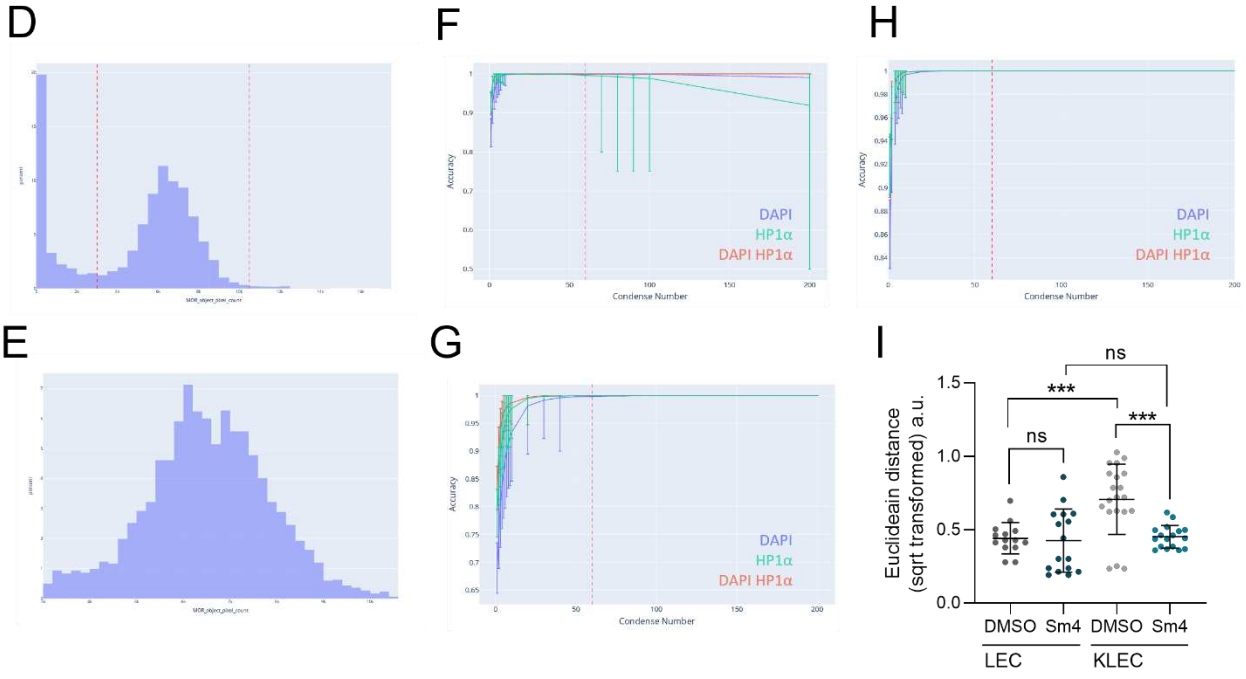
D
F
H
E
G
I
DAPI
HP1α
DAPI HP1α
Condense Number
Accuracy
Euclideain distance
(sqrt transformed) a.u.
ns
***
DMSO
Sm4
LEC
KLEC

Supplementary Figure 5. Related to Fig 4.

A-C. LECs infected with KSHV-BAC16-ΔORF50 (ΔORF50-KLEC) and treated with Sm4 or DMSO for 24h and processed for ATAC-seq. **A.** Clustering and PCA analysis of the ATAC-seq data. **B.** Pearson's analysis of the replicate samples. **C.** Analysis of the ATAC-seq peaks on the KSHV genome in ΔORF50-KLECs treated with DMSO (grey) or Sm4 (red) indicating loss of accessibility sites (blue). **D.** Histogram displaying distribution of all object sizes (pixels) identified during segmentation, objects inside of red-dashed lines are used in analysis and **E.** zoom in on dashed lines in panel D. **F-H.** LEC and KLEC stained with DAPI and anti-HP1α antibody. Line plots showing accuracy measurements versus cell condense number, 95% confidence intervals are shown. **F.** Cell condensation between LEC and KLEC DMSO treatment. **G.** Cell condensation between LEC DMSO vs Sm4 treatment. **H.** Cell condensation between KLEC DMOS vs SM4 treatment **I.** Euclidean distances square root transformed of points from Fig 4L. n = 14 points or more. Statistical significance was determined by one-way ANOVA with Tukey correction for multiple comparisons, ***$p < 0.001$, ns = non-significant.

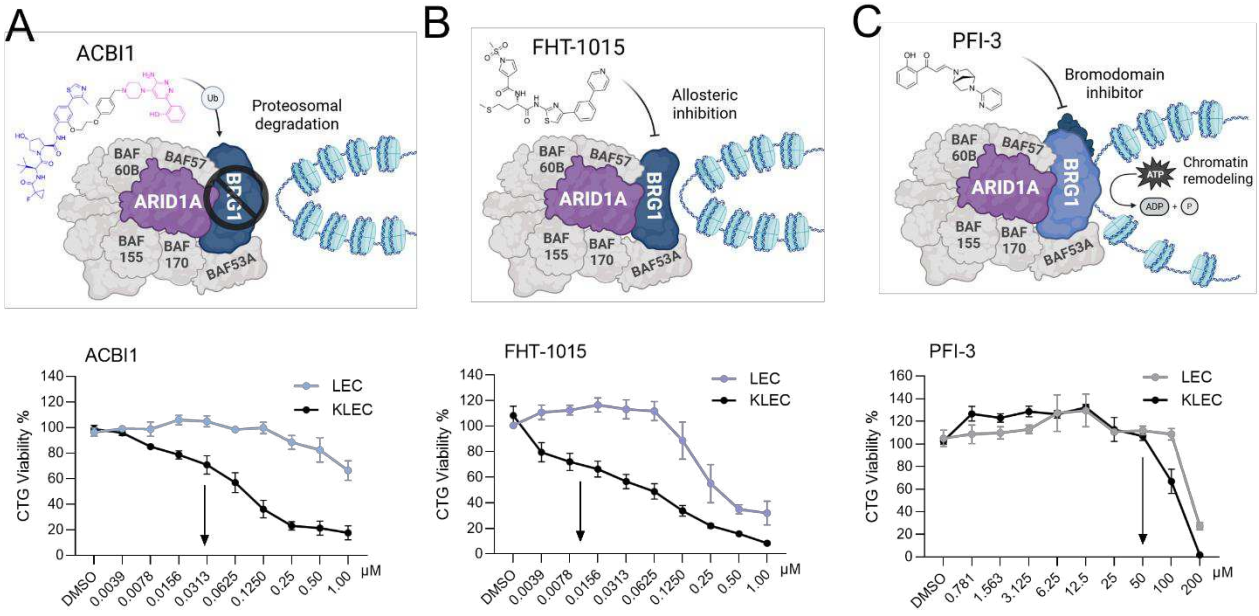
A
ACBI1
Ub
Proteosomal degradation
BAF 60B
BAF57
ARID1A
BRG1
BAF 155
BAF 170
BAF53A
B
FHT-1015
Allosteric inhibition
C
PFI-3
Bromodomain inhibitor
Chromatin remodeling
ATP
ADP
P
ACBI1
FHT-1015
PFI-3
CTG Viability %
LEC
KLEC
DMSO
0.0039
0.0078
0.0156
0.0313
0.0625
0.1250
0.25
0.50
1.00
μM
0.781
1.563
3.125
6.25
12.5
25
50
100
200

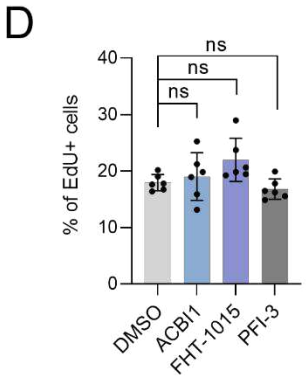
D
% of EdU+ cells
ns
DMSO
ACBI1
FHT-1015
PFI-3

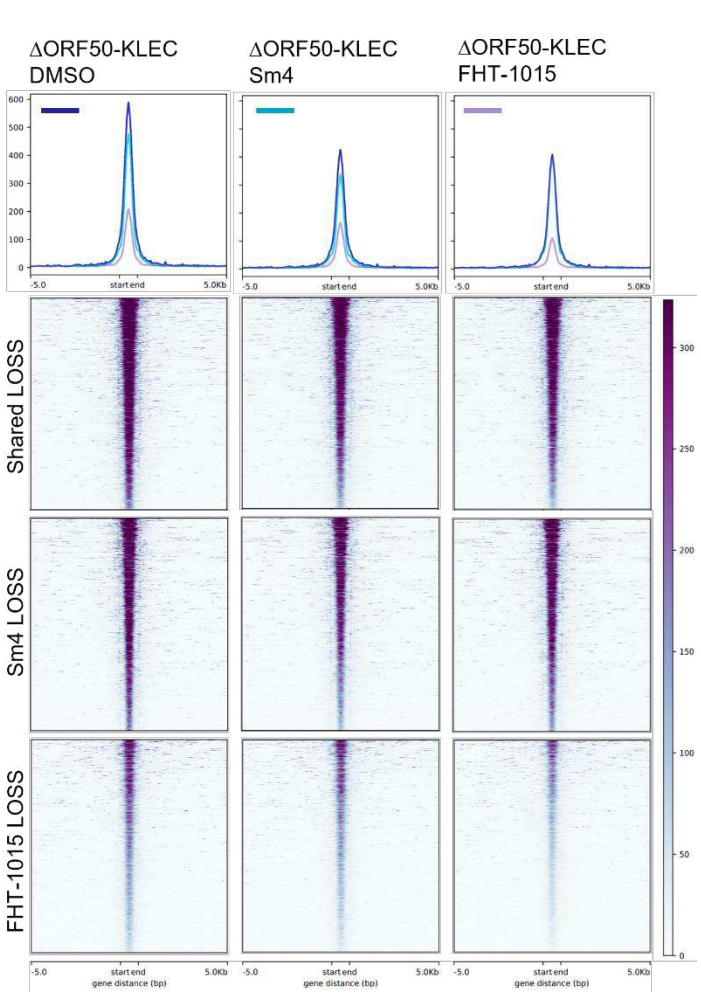
E
ΔORF50-KLEC DMSO
ΔORF50-KLEC Sm4
ΔORF50-KLEC FHT-1015
Shared LOSS
Sm4 LOSS
FHT-1015 LOSS
start end
gene distance (bp)

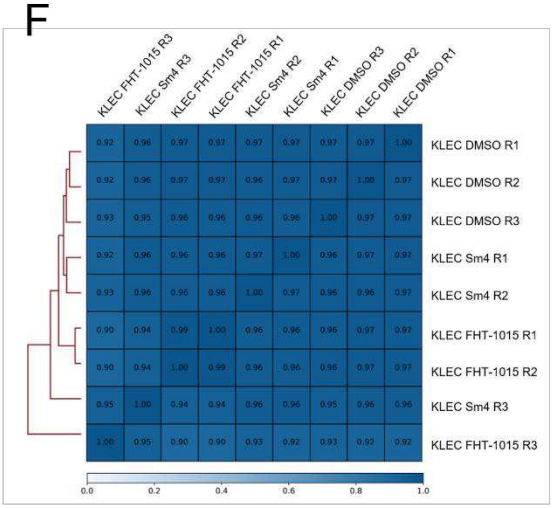
F
KLEC FHT-1015 R3
KLEC Sm4 R3
KLEC FHT-1015 R2
KLEC FHT-1015 R1
KLEC Sm4 R2
KLEC Sm4 R1
KLEC DMSO R3
KLEC DMSO R2
KLEC DMSO R1

Supplementary Figure 6. Related to Fig 5.

A-C. A schematic of the inhibitor mode of action is shown in the top panels. CTG viability assay of uninfected LECs (LEC) or LECs infected with rKSHV.219 (KLEC) for 72h and treated with the indicated, increasing concentrations of BRG1 inhibitors A) ACBI1, B) FHT-1015 and C) PFI-3 (n=3), arrows indicate the selected concentration for following inhibitor assays. **D.** LECs and KLECs were treated with ACBI1, FHT-1015 and PFI-3 and treated with EdU for 4h before subjecting to EdU Click-It, imaged with Opera Phenix 20x and quantified from (n=6 independent replicates, and from each n=100 nuclei). Statistical significance was determined by one-way ANOVA with Dunnett correction for multiple comparisons, ns = non-significant. **E-F.** LECs infected with ΔORF50 and treated with Sm4, FHT-1015, or DMSO for 72h and processed for ATAC-seq. **E.** Peaks and heatmaps of the top 1000 genomic regions with reduced overall accessibility (dark blue maps) showing shared (dark blue line), unique to Sm4 (turquoise) and unique to FHT-1015 (purple) loss sites. **F.** Pearson's analysis of the replicate (n = 3) samples. ns = non-significant.

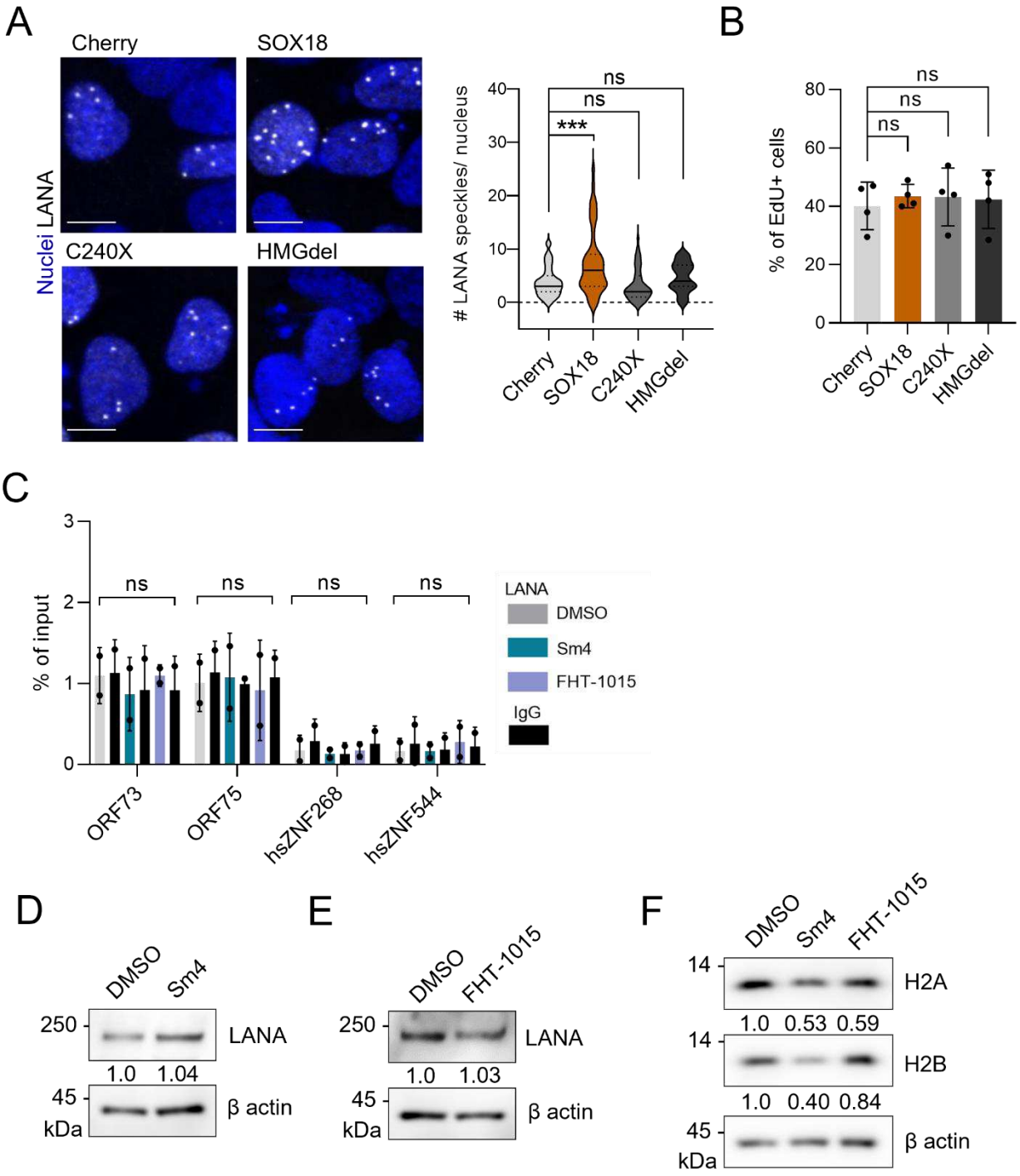
A
Cherry
SOX18
C240X
HMGdel
Nuclei LANA
LANA speckles/ nucleus
ns
ns
***
Cherry
SOX18
C240X
HMGdel
B
% of EdU+ cells
ns
ns
ns
Cherry
SOX18
C240X
HMGdel
C
% of input
ns
ns
ns
ns
ORF73
ORF75
hsZNF268
hsZNF544
LANA
DMSO
Sm4
FHT-1015
IgG
D
DMSO
Sm4
250
LANA
1.0 1.04
45
kDa
β actin
E
DMSO
FHT-1015
250
LANA
1.0 1.03
45
kDa
β actin
F
DMSO
Sm4
FHT-1015
14
H2A
1.0 0.53 0.59
14
H2B
1.0 0.40 0.84
45
kDa
β actin

Supplementary Figure 7. Related to Fig 6.

A. Confocal images of LANA speckles (above panel) and quantified (bottom panel) as a number of nuclear speckles (n=50 nuclei), nuclei were counterstained with Hoechst (33342), scale bar is 10μm. **B.** HeLa cells expressing SOX18wt or the indicated mutants treated with EdU for 2h before fixing and subjected to EdU Click-It, imaged and quantified (n=4 independent replicates, and from each n=100 nuclei). **C-E.** LECs infected with rKSHV.219 for 72h were treated with DMSO, Sm4 or FHT-1015 for 24h and C) subjected to ChIP-PCR using anti-LANA and IgG antibodies for viral and human non-LANA binding control sites (n=2), and D-E) immunoblotted for LANA and β-actin as a loading control for normalization. **F.** KLECs treated for 72h and immunoblotted for H2A and H2B and quantified as in D-E. Statistical significance was determined by one-way ANOVA with either Dunnett or Tukey correction for multiple comparisons, ***$p < 0.001$, ns = non-significant.
